# Apolipoprotein E and clusterin inhibit the early phase of amyloid-β aggregation in an in vitro model of cerebral amyloid angiopathy

**DOI:** 10.1186/s40478-019-0662-1

**Published:** 2019-01-28

**Authors:** Yoshinori Endo, Kazuhiro Hasegawa, Ryo Nomura, Hidetaka Arishima, Ken-ichiro Kikuta, Taro Yamashita, Yasuteru Inoue, Mitsuharu Ueda, Yukio Ando, Mark R. Wilson, Tadanori Hamano, Yasunari Nakamoto, Hironobu Naiki

**Affiliations:** 1 Second Department of Internal Medicine, University of Fukui, Fukui, 910-1193 Japan; 2 Department of Molecular Pathology, Faculty of Medical Sciences, University of Fukui, Fukui, 910-1193 Japan; 3 Department of Neurosurgery, University of Fukui, Fukui, 910-1193 Japan; 4 Department of Neurology, Graduate School of Medical Sciences, University of Kumamoto, Kumamoto, 860-8556 Japan; 5 School of Chemistry and Molecular Bioscience, Illawarra Health and Medical Research Institute (IHMRI), and Molecular Horizons Research Institute, University of Wollongong, Wollongong, New South Wales 2522 Australia; 6 Department of Aging and Dementia, University of Fukui, Fukui, 910-1193 Japan

**Keywords:** Cerebral amyloid angiopathy, Amyloid-β, Proteome analysis, Apolipoprotein E, Clusterin, Intramural periarterial drainage, Extracellular chaperone

## Abstract

Sporadic cerebral amyloid angiopathy (CAA) is characterized by cerebrovascular amyloid-β (Aβ) deposition, which leads to lobar hemorrhage and dementia. Biological molecules affecting the development of CAA have not been fully characterized. In this study, we performed proteome analysis of biopsied leptomeningeal and cortical vessels obtained from 6 CAA patients and 5 non-CAA patients who underwent surgery for large lobar hemorrhages. We found that 6 proteins, including Aβ, apolipoprotein E (apoE), clusterin (CLU), albumin, complement C4 and vitronectin were significantly upregulated in the vessels of CAA patients as compared to non-CAA patients. ApoE and CLU were found in all CAA patients. We next examined the effects of apoE and CLU on the early phase of Aβ aggregation, using a simple yet powerful in vitro model of CAA, which recapitulates the intramural periarterial drainage pathway model. We found that physiological concentrations of apoE and CLU delayed the initiation time of amyloid growth kinetics in a concentration-dependent manner. These data indicate that apoE and CLU may act as extracellular chaperones to inhibit Aβ amyloid deposition in CAA.

## Introduction

Sporadic cerebral amyloid angiopathy (CAA) is characterized by amyloid-β (Aβ) deposition in the cortical and leptomeningeal arteries, which leads to lobar hemorrhage, infarction, encephalopathies and dementia [40, 41]. Recently, several groups reported that various proteins are deposited with Aβ in the vessel walls of postmortem, autopsied CAA patients [14, 17, 20]. Apolipoprotein E (apoE), clusterin (CLU, also called apolipoprotein J. apoJ), serum amyloid-P component, tissue inhibitor of metalloproteinases-3, sushi repeat-containing protein 1 (SRPX1) and other proteins are the major Aβ-associated proteins in the vessel walls of CAA patients. However, the effects of these proteins on the development of CAA are not clear except for SRPX1, which may enhance the cerebrovascular degeneration induced by Aβ [17].

In this study, we first performed proteome analysis of biopsied leptomeningeal and cortical vessels obtained from 6 CAA patients and 5 non-CAA patients who underwent surgery for large lobar hemorrhages [18]. We found that expression of apoE and CLU is significantly increased in the vessels of acute-onset, symptomatic CAA patients as compared to non-CAA patients. Next, we examined the effects of apoE and CLU on the early phase of Aβ aggregation, using a powerful, physiologically relevant in vitro model of CAA [10]. This model was previously established to test the hypothesis that intramural periarterial drainage (IPAD) flow carries Aβ to the vascular basement membrane (BM), which may trap Aβ and induce amyloid fibril formation in vivo [1, 22].

## Materials and methods

### Materials

Aβ(1–40) (code 4307-v, trifluoroacetic salt, lyophilized from dimethyl sulfoxide solution) was purchased from Peptide Institute Inc. (Osaka, Japan). Human serum albumin (HSA) (code 70024–90-7, A8763) was purchased from Sigma. Matrigel (Phenol Red free, code 356237) was purchased from Becton-Dickinson and Co. (NJ, USA). NHS-activated Sepharose 4 Fast Flow (code 17–0906-01) was purchased from GE Healthcare UK Ltd. Recombinant human apolipoprotein E3 (apoE3) (code 010–20261) and apoE4 (code 017–20271) were purchased from Wako (Osaka, Japan). Human CLU was purified from human serum as described previously [29].

### Patients and specimens for proteome analysis

We recently reported the prevalence of CAA in patients who underwent surgery in our hospital for large lobar hemorrhages, i.e., supratentorial bleeding expanding from the cerebral cortex to subcortical white matter [18]. To diagnose CAA, we examined biopsied cortical tissues around hematomas with Congo-red and anti-Aβ staining. The same biopsied tissue was used for a series of histopathological and immunohistochemical staining to diagnose CAA and for the subsequent proteome analysis. We examined a cohort of 24 CAA patients and 5 non-CAA patients. Of 24 CAA patients, 16 patients (66.7%) had severe (Grade 4) CAA based on the pathological grading system for CAA developed by Greenberg et al. [9].

From this cohort, we selected 6 CAA patients (all Grade 4) and 4 non-CAA patients for which sufficient amounts of pathological specimens were available for the subsequent proteome analysis (Table 1). To increase the patient number, we added one additional non-CAA patient who underwent surgery in our hospital (B-1 in Table 1).

**Table 1:** Profiles of cases of CAA and non-CAA patients analysed by LMD-LC-MS/MS

Number	Group	Age	Sex	Lesion of brain hemorrhage	Amyloid grading scale^a^	Hypertension and medication	Anticoagulants or antiplatelets	Microbleeding at MRI (T2*)	Patient number in Table 3 of [18]
A-1	CAA	66	F	R temporo-parietal	4	No	No	negative	3
A-2	CAA	80	F	L temporo-parietal	4	Yes, medication	No	NA	4
A-3	CAA	79	F	L frontal	4	No	No	NA	5
A-4	CAA	74	F	R frontal	4	Yes, medication	No	positive	6
A-5	CAA	71	F	L frontal	4	Yes, medication^b^	No	positive	7
A-6	CAA	63	F	L parietal	4	No	No	negative	12
B-1	non-CAA	83	F	R putamen and frontal	0	Yes, no medication	No	negative	Not included
B-2	non-CAA	67	M	R fronto-parietal	0	Yes, no medication	No	negative	25
B-3	non-CAA	75	M	R temporo-parietal	0	No	No	NA	26
B-4	non-CAA	61	M	R frontal	0	No	No	negative	28
B-5	non-CAA	68	M	R frontal	0	Yes, medication	No	NA	27

*M* male, *F* female, *R* right, *L* left, *NA* not applicable

^a^Pathological grading system for CAA by Greenberg SM et al. [9]

^b^Self-withdrawal 2 years before onset

### Protein extraction and proteome analysis

Protein extraction and proteome analysis were performed with liquid chromatography-tandem mass spectrometry (LC-MS/MS), essentially as described elsewhere [17]. Briefly, 4 μm thick slices of formalin-fixed and paraffin-embedded brain biopsy samples were placed on membrane slides (Leica Microsystems, Wetzlar, Germany). Sections were air-dried and then melted, deparaffinized, and stained with Congo red combined with nuclear counterstaining with hematoxylin. In the CAA group, Congo red-positive leptomeningeal and cortical vessels, which were identified using the bright-field setting, were isolated via laser capture microdissection (LCM) (LMD7000; Leica Microsystems, Wetzlar, Germany) (Table 1), then analysed using nano-flow reversed-phase LC–MS/MS (LTQ Velos Pro; Thermo Fisher Scientific). In the non-CAA group, leptomeningeal and cortical vessels, which were identified using the bright-field setting, were isolated via LCM. In both groups, we didn’t discriminate arteries from veins. The relative abundances of the identified molecules were obtained using the normalized spectral abundance factor (NSAF) [27] (Table 2).

**Table 2:** Proteins in the cerebral blood vessels of CAA patients vs. non-CAA patients

Accession number	Protein	CAA (*n* = 6)^a^		Non-CAA (*n* = 5) ^a^		*p* value*
		% Detection	NSAF_CAA_	% Detection	NSAF_non-CAA_	
**P02649**	**Apolipoprotein E**	**100**	**0.1259**	**20**	**0.0006**	**0.004**
**P05067**	**Amyloid beta A4 protein**	**100**	**0.0685**	**0**	**ND**	**0.004**
**P10909**	**Clusterin**	**100**	**0.039**	**20**	**0.0015**	**0.004**
**P02768**	**Serum albumin**	**100**	**0.046**	**100**	**0.0154**	**0.017**
P08123	Collagen alpha-2(I) chain	100	0.0031	80	0.0019	0.329
P68871	Hemoglobin subunit beta	100	0.1695	100	0.249	0.662
P69905	Hemoglobin subunit alpha	100	0.0778	100	0.0718	0.792
**P04004**	**Vitronectin**	**83**	**0.0085**	**20**	**0.0004**	**0.03**
**P0C0L4**	**Complement C4-A**	**83**	**0.0032**	**20**	**0.0001**	**0.03**
P08670	Vimentin	83	0.0207	80	0.0159	0.247
P41222	Prostaglandin-H2 D-isomerase	67	0.013	40	0.0021	0.177
Q15149	Plectin	67	0.0006	40	0.0001	0.177
Q8IYA6	Cytoskeleton-associated protein 2-like	67	0.0147	80	0.006	0.537
P06727	Apolipoprotein A-IV	50	0.0096	0	ND	0.177
Q70EL1	Inactive ubiquitin carboxyl-terminal hydrolase 54	50	0.0035	0	ND	0.177
P11047	Laminin subunit gamma-1	50	0.0014	0	ND	0.177
P02042	Hemoglobin subunit delta	50	0.0313	80	0.0735	0.247
Q8N413	Solute carrier family 25 member 45	50	0.0102	20	0.0011	0.329
P35625	Metalloproteinase inhibitor 3	50	0.0084	20	0.0009	0.329
P07437	Tubulin beta chain	50	0.0061	40	0.0018	0.429
P12814	Alpha-actinin-1	50	0.0015	40	0.0005	0.429
P14136	Glial fibrillary acidic protein	50	0.0265	60	0.0116	0.662
Q9BQE3	Tubulin alpha-1C chain	50	0.0051	60	0.0018	0.662
A6NNT2	Putative uncharacterized protein C16orf96	50	0.004	60	0.0011	0.662
P04350	Tubulin beta-4A chain	50	0.0064	80	0.0045	0.792
P98160	Basement membrane-specific heparan sulfate proteoglycan core protein	50	0.0004	60	0.0004	1

Proteins which show the % detection to be ≥50% in CAA patients are listed. % Detection means the relative number of patients positive for each protein in both CAA and non-CAA patients

*ND* not detected

*The Mann-Whitney *U* test was used for comparisons between NSAF_CAA_ and NSAF_non-CAA_ values

^a^Protein abundance values were estimated using NSAF (normalized spectral abundance factor) normalizaiton

Boldface highlights the proteins which were significantly upregulated in the cerebral blood vessels of CAA patients as compared to non-CAA patients

### Kinetic analysis of the seeded aggregation of Aβ(1–40) amyloid fibrils

In this paper, we used only Aβ(1–40) because Aβ(1–40) is the predominant Aβ species deposited in the vessels of CAA patients [40, 41]. Aβ(1–40) amyloid fibrils (fAβ(1–40)) were first formed by incubating 1.0 ml of the reaction mixture containing 50 μM Aβ(1–40), 50 mM sodium phosphate, pH 7.5, 100 mM NaCl phosphate buffered saline (PBS), and 0.05% NaN_3_ at 37 °C for 1 week. Subsequently, a reaction mixture containing 2.5 μg/ml fAβ(1–40), 5 μM Aβ(1–40), 0–0.5 μM apoE3 or 0–1.0 μM CLU, 0.3 mg/ml (4.5 μM) HSA, PBS, and 5 μM thioflavin T (ThT) was incubated at 37 °C without shaking in a 96-well plate (code HSP9666, Bio Rad, USA) sealed with a sealing film (code 676070, Greiner Bio-One GmbH, Frickenhausen, Germany). ThT fluorescence was measured every 5 min for 2 h using a Safire2 microplate fluorometer (TECAN, Austria) with excitation at 445 nm and emission at 490 nm.

### Analysis of the effects of ApoE and CLU on the length of the lag phase of Aβ(1–40) amyloid aggregation

**Fig. 1:**
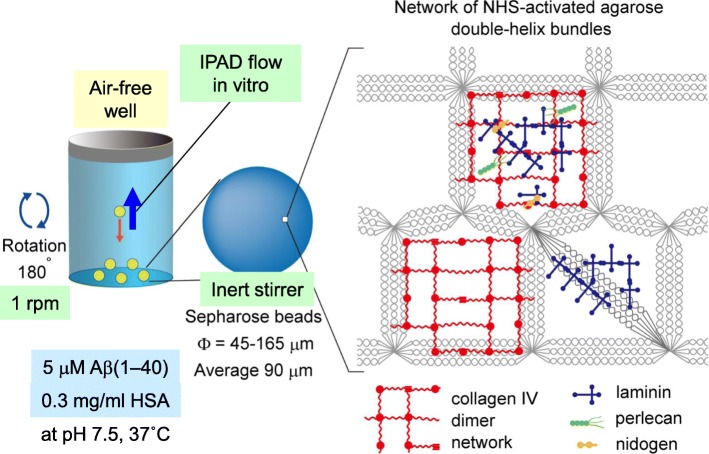
Schematic representation of the in vitro model of CAA used in this study. Sepharose 4 Fast Flow beads are highly cross-linked 4% agarose beads with a mean diameter of 90 μm. The pore sizes of the beads may be similar to the sizes of BM components, resulting in the conjugation of BM components onto the surface of beads. During the rotation, beads are exposed to the relative countercurrent of the reaction mixture, which mimics intramural periarterial drainage (IPAD) flow in vitro and enhances the interaction of Aβ with surface-bound BM components leading to Aβ aggregation. HSA: human serum albumin (modified from Fig. 6 of [10])

In this paper, we utilized a previously established powerful in vitro model of CAA [10] to analyse the effects of apoE and CLU on the length of the lag phase of Aβ(1–40) amyloid aggregation, essentially as described in [10]. Briefly, we reconstituted an artificial BM on the surface of NHS-activated Sepharose 4 Fast Flow beads by conjugating Matrigel to their surface (Fig. 1). Matrigel-coated beads were then incubated with 5 μM Aβ(1–40), 0.3 mg/ml (4.5 μM) HSA, PBS, 0.05% NaN_3_ (PBS-NaN_3_), 5 μM ThT, and 0–0.5 μM apoE3/E4 or 0–1.0 μM CLU at 37 °C in a clear microtiter plate module (Nunc, F8 Immuno module, Maxisorp, code: 468667) in which the air water interface was completely removed. The plate was gently rotated at 1 rpm. As these beads slowly sink from the top to the bottom of a well, their surfaces are exposed to the relative countercurrent of the reaction mixture, which mimics the IPAD flow in vitro.

The ThT-reactive aggregates in the microtiter wells were visualized with a fluorescence microscope (MVX10, Olympus Corporation, Tokyo, Japan) equipped with CFP filter sets (excitation 425–445 nm, emission 460–510 nm). Then the fluorescence images recorded for 1 s with a DP 72 digital camera (Olympus Corporation, Tokyo, Japan). We chose the first time point at which ThT-reactive aggregates were detected as the initiation time for fibril growth kinetics. The preceding lag phase is the time during which nuclei, on-pathway oligomers and protofibrils are successively formed prior to the subsequent rapid fibril growth phase. In most cases, one observer (Y.E.) mainly determined the initiation time by visual inspection of recorded images. In the specific cases of data shown in Figs. 4, 5 and 6, another observer (R.N.) re-determined the initiation time and similar results were obtained (data not shown).

To monitor Aβ(1–40) amyloid formation in a conventional way, we also measured the ThT fluorescence with a Safire2 microplate fluorometer (TECAN, Austria). For the kinetic analysis, data obtained with a fluorescence microscope was used because microscopic detection of fluorescent spots on the Sepharose-beads often preceded the increase in fluorescence measured by fluorometry by 12 to 24 h (data not shown).

**Fig. 2:**
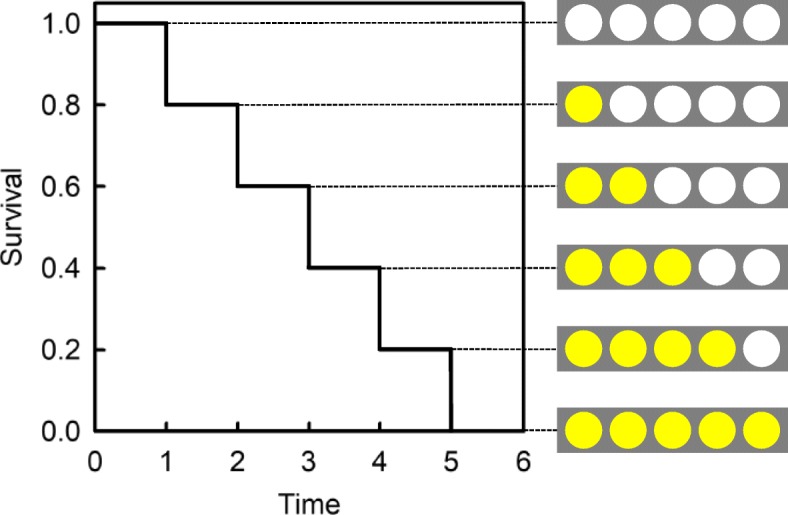
Kaplan-Meier survival method used to evaluate the duration of the lag phase of Aβ aggregation. The kinetics were analysed by the Kaplan-Meier survival method using the initiation time of amyloid growth kinetics as the event of interest. Aβ(1–40) was incubated with Matrigel-coated Sepharose beads as described in Materials and Methods. The appearance of ThT-reactive aggregates was monitored with a fluorescence microscope. The first time at which ThT-reactive aggregates were detected was set as the initiation time of amyloid growth. When ThT-reactive aggregates are detected in 1 well of 5, the “survival rate” is recorded as having decreased from 1.0 to 0.8. Similarly, if ThT-reactive aggregates are then detected in 1 of the remaining 4 wells, then the “survival rate” is recorded as having decreased from 0.8 to 0.6. Thus, the slower the kinetics of early phase Aβ aggregation, the more time is required to reach a “survival” value of zero. This model was used to evaluate the concentration-dependent effects of apoE and CLU on the kinetics of early phase Aβ aggregation

We then measured the duration of the lag phase for each well using the Kaplan-Meier survival method and the initiation time of amyloid growth kinetics as the event of interest (Fig. 2). In this assay, the reaction mixture in which ThT-reactive aggregates have not yet been detected is considered as “surviving”. Thus, the survival rate corresponds to the percentage of the reaction mixtures in which ThT-reactive aggregates have not yet been detected. The statistical significance was compared by the log rank test, followed by pair to pair multiple comparisons using the Holm-Sidak method. For the calculation, we excluded any wells in which air bubbles emerged before the detection of ThT-reactive aggregates. The survival analysis was performed with SigmaPlot 12 (Systat Software, Inc. CA). The differences between the two groups were considered significant if *P* values were less than 0.05.

## Results

### Profiles of CAA and non-CAA patients

Table 1 shows the profiles of the CAA and non-CAA patients analysed by LCM-LC-MS/MS. The 6 CAA patients were from 63 to 80 years old (mean ± SD 72.2 ± 6.9). Compatible with the female predominance in CAA patients [41], all 6 patients were female and had grade 4 CAA. Three of the 6 patients had hypertension and took antihypertensive agents. No patients took anticoagulant or antiplatelet drugs. Two patients were positive for strictly lobar microbleeding as evaluated by T2*-weighted magnetic resonance imaging (MRI). The ages of the 5 non-CAA patients were from 61 to 83 years old (mean ± SD 70.8 ± 8.4). Four patients were male, and 1 patient was female. We found no female predominance in non-CAA patients. Three of the 5 patients had hypertension and only 1 took antihypertensive agents. Thus, it is reasonable to consider that for these 3 patients, the etiology of hemorrhage may be hypertension. As for the remaining 2 patients, although we excluded CAA as an etiology of hemorrhage, we did not definitely identify the etiology of hemorrhage. No patients took anticoagulant or antiplatelet drugs. No patients were positive for microbleeding as evaluated by T2*-weighted MRI.

### Proteome analysis

As shown in Table 2, 6 proteins, including Aβ, apoE, CLU, albumin, complement C4 and vitronectin were significantly upregulated in the cerebral blood vessels of CAA patients as compared to non-CAA patients. Aβ was found only in CAA patients. Albumin was found in all patients of CAA and non-CAA groups. ApoE and CLU were found in all patients of the CAA group but were found in only 1 patient of the non-CAA group (20%). Both apoE and CLU are representative amyloid signature proteins [2]. Thus, in the following study, we analysed the effects of apoE and CLU on Aβ amyloid formation in two different in vitro systems.

### The effects of apoE and CLU on the seeded aggregation of Aβ(1–40) amyloid fibrils

**Fig. 3:**
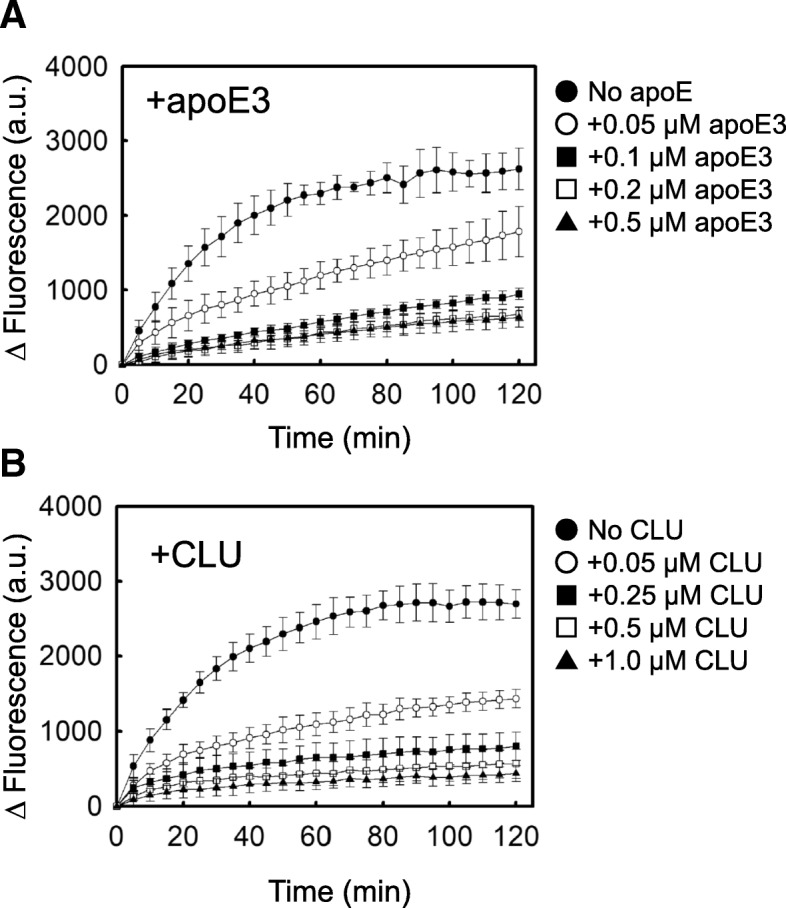
Effects of apoE and CLU on the seeded aggregation of fAβ(1–40) in vitro. **a** The reaction mixture containing 2.5 μg/ml fAβ(1–40), 5 μM Aβ(1–40), 0 (●), 0.05 (○), 0.1 (■), 0.2 (□), 0.5 μM (▲) apoE3, 0.3 mg/ml HSA, PBS, and 5 μM ThT was incubated at 37 °C without shaking in a 96-well plate. **b** The reaction mixture containing 2.5 μg/ml fAβ(1–40), 5 μM Aβ(1–40), 0 (●), 0.05 (○), 0.25 (■), 0.5 (□), 1.0 μM (▲) CLU, 0.3 mg/ml HSA, PBS, and 5 μM ThT was incubated at 37 °C without shaking in a 96-well plate. ThT fluorescence was monitored as described in Materials and Methods. Points and bars represent mean + S.D. of 6 replicates. This data is representative of three independent experiments

We first used the conventional in vitro experimental system with an air-water interface to examine the effects of apoE and CLU on the seeded aggregation of Aβ(1–40) amyloid. In the absence of apoE and CLU, ThT fluorescence increased rapidly with no lag time to reach a plateau at around 2 h after initiation of the reaction (Fig. 3), consistent with the first-order kinetic model of amyloid fibril growth in vitro [25]. As shown in Fig. 3, both apoE and CLU concentration-dependently inhibited the formation of Aβ amyloid in this system.

### The effects of apoE and CLU on the duration of the lag phase of Aβ aggregation in an in vitro model of CAA

**Fig. 4:**
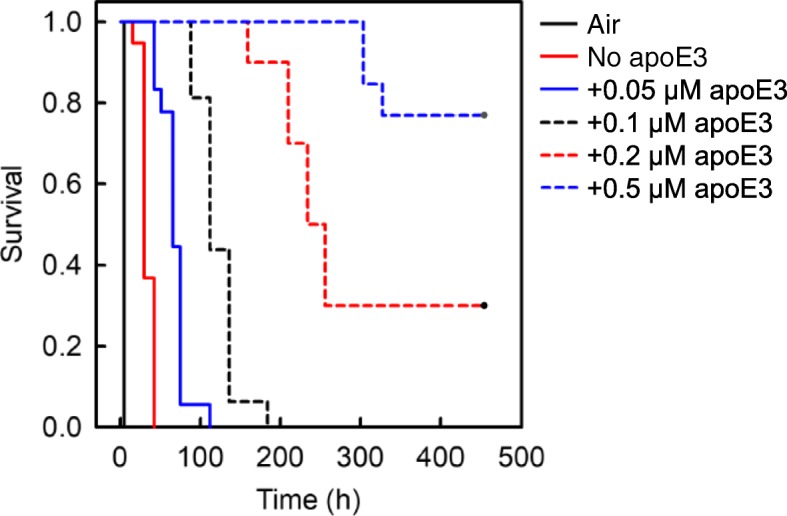
Effect of apoE3 on the kinetics of early phase aggregation of Aβ(1–40). The reaction mixture containing 5 μM Aβ(1–40), 10 μl suspensions of Matrigel-coated beads, 0.3 mg/ml HSA, PBS-NaN_3_, 5 μM ThT, and 0 to 0.5 μM apoE3 was incubated at 37 °C with rotation at 1 rpm in the absence of an air-water interface (30 replicate wells in each case). As a positive control, the reaction mixture containing 5 μM Aβ(1–40), 0.3 mg/ml HSA, PBS-NaN_3_, and 5 μM ThT was also incubated at 37 °C with rotation at 1 rpm in the presence of an air-water interface (12 replicate wells) (Air). The kinetics of early phase aggregation was analysed by the Kaplan-Meier survival method using the initiation time of fibril growth kinetics as the event of interest (see Fig. 2). The *P* value was less than 0.05 between all combinations. This data is representative of three independent experiments

**Fig. 5:**
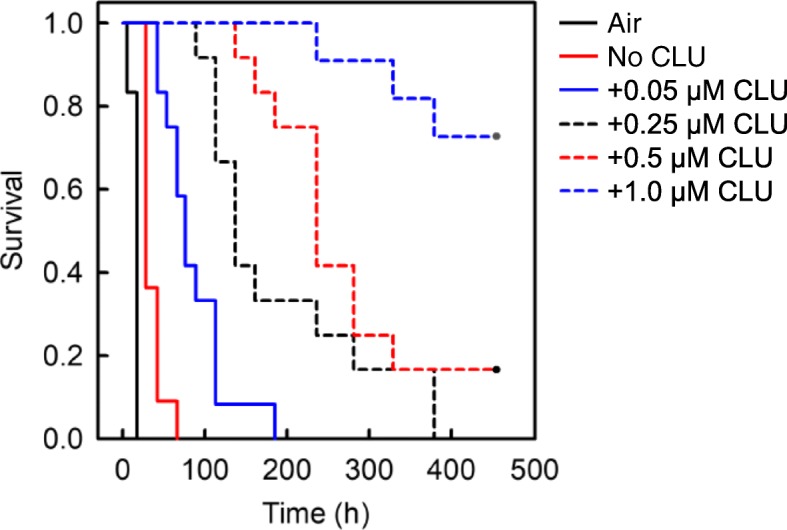
Effect of CLU on the kinetics of early phase aggregation of Aβ(1–40). The reaction mixture containing 5 μM Aβ(1–40), 10 μl suspensions of Matrigel-coated beads, 0.3 mg/ml HSA, PBS-NaN_3_, 5 μM ThT, and 0 to 1.0 μM CLU was incubated at 37 °C with rotation at 1 rpm in the absence of an air-water interface (18 replicate wells in each case). As a positive control, the reaction mixture containing 5 μM Aβ(1–40), 0.3 mg/ml HSA, PBS-NaN_3_, and 5 μM ThT was also incubated at 37 °C with rotation at 1 rpm in the presence of an air-water interface (18 replicate wells) (Air). The kinetics of early phase aggregation was analysed by the Kaplan-Meier survival method using the initiation time of fibril growth kinetics as the event of interest (see Fig. 2). The P value was less than 0.05 between all combinations except for between 0.25 and 0.5 μM CLU. This data is representative of three independent experiments

**Fig. 6:**
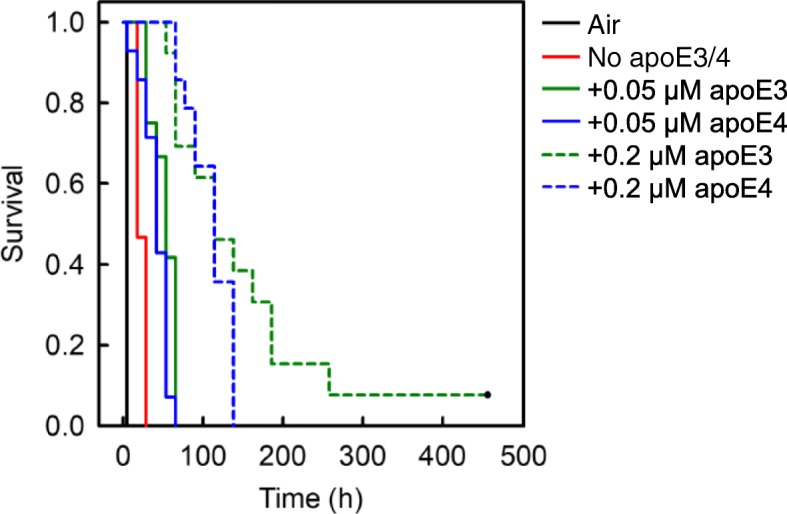
Comparison of the effect of apoE3 with apoE4 on the kinetics of early-phase Aβ(1–40) aggregation. The reaction mixture containing 5 μM Aβ(1–40), 10 μl suspensions of Matrigel-coated beads, 0.3 mg/ml HSA, PBS-NaN_3_, 5 μM ThT, and 0 to 0.2 μM apoE3 or apoE4 was incubated at 37 °C with rotation at 1 rpm in the absence of an air-water interface (18 replicate wells in each case). As a positive control, the reaction mixture containing 5 μM Aβ(1–40), 0.3 mg/ml HSA, PBS-NaN_3_, and 5 μM ThT was also incubated at 37 °C with rotation at 1 rpm in the presence of an air-water interface (18 replicate wells) (Air). The kinetics of early phase aggregation was analysed by the Kaplan-Meier survival method using the initiation time of fibril growth kinetics as the event of interest (see Fig. 2). The P value was less than 0.05 for all pair-wise comparisons except those between apoE3 and apoE4 when both were at either 0.05 μM or 0.2 μM. This data is representative of three independent experiments

As shown in Fig. 4, apoE3 delayed the initiation time of fibril growth kinetics in a concentration-dependent manner, indicating that apoE3 inhibited early phase Aβ aggregation. Importantly, apoE3 significantly inhibited the early phase aggregation of Aβ when at a concentration that is physiological in the cerebrospinal fluid (CSF) (1.8–4.0 μg/ml or 0.05–0.12 μM) [28]. CLU also concentration-dependently delayed the initiation time of fibril growth kinetics (Fig. 5), significantly inhibiting the early phase of Aβ aggregation when at a concentration that is physiological in CSF (3.5–5.7 μg/ml or 0.07–0.11 μM) [33]. As shown in Fig. 6, the same concentrations of apoE3 and E4 exhibited no significant isoform-dependent difference in their ability to inhibit the early phase aggregation of Aβ.

We previously reported that under these same experimental conditions, 0.1–1.0 mg/ml (1.5–15.1 μM) HSA has no significant effect on the early phase of Aβ aggregation [10]. Thus, we conclude that apoE and CLU specifically and concentration-dependently inhibit the early phase of Aβ aggregation.

## Discussion

In this study, we first performed proteome analysis of the Aβ-deposited leptomeningeal and cortical vessels (Tables 1 and 2). To the best of our knowledge, this is the first report of the proteome analysis of vessels biopsied from living symptomatic, clinical CAA patients. In previously published reports, proteome analysis was performed with leptomeningeal vessels obtained from postmortem, autopsied cases, in which Aβ deposition was histopathologically confirmed [14, 17, 20]. Although CAA is known to be more severe in posterior brain regions compared to anterior brain regions [41] and postmortem proteome analysis was performed using the vessels derived from occipital lobes [14, 17, 20], frontal lobes were involved in 3 CAA patients and parietal and temporal lobes were involved in other 3 CAA patients (Table 1). This difference in the brain regions from which the vessels were obtained may affect the results of proteome analysis (Table 2 vs. [14, 17, 20]). For example, this may explain why we found no significant upregulation of tissue inhibitor of metalloproteinases-3 (Table 2), in contrast to the finding of Manousopoulou et al. [20]. Additionally, the discrepancy in male/female ratio between CAA and non-CAA patients (male/female: 0/6 and 4/1, respectively) may also affect the results of our proteome analysis. Both apoE and CLU are representative signature proteins in various types of systemic amyloidosis [2]. Thus, it is worth noting that apoE and CLU are representative Aβ-associated proteins in the vessel walls of clinical CAA cases (Table 2), as well as in those of pathological CAA cases [14, 17, 20].

Carare and coworkers proposed the IPAD pathway model [1, 22, 34]. This model constitutes that instead of the conventional lymphatics, interstitial fluid and solutes are drained from the brain parenchyma to cervical lymph nodes along BMs in the walls of cerebral capillaries and tunica media of leptomeningeal arteries. Through this pathway, Aβ peptides, especially Aβ(1–40) produced by cortical neurons are carried away from the brain parenchyma [1, 22]. Reduced Aβ trafficking through this pathway may result in the aggregation of Aβ amyloid in the cerebrovascular BMs, leading to the manifestation of pathological as well as clinical CAA [41]. However, the driving mechanisms of this pathway and the molecules affecting the trafficking and aggregation of Aβ in this pathway are not fully understood [41, 43]. We previously established a simple yet powerful in vitro model of CAA, which recapitulates the IPAD flow draining Aβ and other solutes, as well as the vascular BM as a scaffold for Aβ aggregation in vitro [10]. To the best of our knowledge, this is the first, and the only in vitro system which recapitulates the pathogenesis of CAA in a physiologically relevant manner (Fig. 1). First, we completely removed the air-water interface, which is a hydrophobic-hydrophilic interface that potently induces protein denaturation and amyloid formation [21]. Complete removal of the air-water interface makes it possible to detect weak effects of BM molecules on the induction of amyloid formation in vitro. Second, we reduced Aβ concentration as low as possible (5 μM) to inhibit the spontaneous aggregation of Aβ in the reaction mixture. We also added HSA (4.5 μM) to mimic the CSF environment. Finally, we reproduced the IPAD flow in vitro by gently rotating the plate at 1 rpm. As Matrigel-coated beads slowly sink from the top to the bottom of a well, their surfaces are exposed to the relative countercurrent of the reaction mixture to induce the interaction of Aβ with BM molecules. Using this model, we have demonstrated that apoE and CLU inhibit the early phase aggregation of Aβ in vitro (Figs. 4 and 5).

Although apoE is considered as a key player in the pathogenesis of Alzheimer’s disease (AD) and CAA [16, 32], the effects of apoE on the aggregation of Aβ in vitro and in vivo are controversial. While some groups reported that apoE accelerates Aβ fibril formation in vitro [31, 35], we and other groups reported that apoE inhibits Aβ aggregation in vitro [6, 8, 15, 24, 37]. These opposite effects may be partly due to the difference in the concentrations of Aβ used in each experiment. We previously reported that apoE may inhibit or enhance Aβ amyloid fibril formation in a concentration-dependent manner [24]. When 50 μM Aβ(1–40) was incubated with 50–500 nM apoE, apoE dose-dependently inhibited Aβ amyloid fibril formation. In contrast, when 300 μM Aβ(1–40) was incubated with 3 μM apoE, apoE slightly enhanced Aβ aggregation. Similarly, while some groups reported that apoE promotes Aβ amyloid deposition in vivo [12], other groups showed that apoE delays Aβ amyloid deposition in vivo [4, 7, 13, 15, 32]. LaDu and coworkers produced EFAD mice, which are a tractable familial AD-transgenic (FAD-Tg) mouse model expressing human *APOE* rather than mouse *APOE* [32]. Consistent with our data (Fig. 4), they showed that introduction of human *APOE* to EFAD mice delays extracellular Aβ accumulation (not only plaque deposition but also CAA) from ~ 2 to 6 months compared with the control 5xFAD mice expressing mouse *APOE* [32]. They suggested that the mouse apoE is structurally and functionally distinct from human apoE.

The pathogenesis of AD and CAA is affected by apoE isoform-dependently [16, 32]. Robust data confirmed that ε4 allele of *APOE* is not only the risk factor of AD but also that of nonhemorrhagic-type CAA [3, 16, 32, 40]. In contrast, while the ε2 allele of *APOE* is protective for the manifestation of AD, it is a risk factor of hemorrhagic-type CAA [3, 16, 32, 40]. Tai et al. reported that the ability of human *APOE* to delay the extracellular Aβ accumulation in EFAD mice was in the order of 5xFAD < E4FAD < E3FAD ≤ E2FAD [32]. Consistent with this in vivo observation, Hori et al. reported that the in vitro conversion of Aβ protofibrils to fibrils progressed more slowly upon co-incubation with apoE2 or apoE3 as compared to the case with apoE4 [15]. In contrast, we found that the inhibitory effect of apoE3 on the kinetics of early phase Aβ aggregation was not significantly different from that of apoE4 (Fig. 6). It is hypothesized that apoE affects the pathogenesis of AD and CAA through a variety of mechanisms, including the effects on Aβ aggregation, Aβ transport and clearance from the interstitial/cerebrospinal fluid, and cellular metabolism of Aβ [16]. Thus, the linkage of ε4 allele of *APOE* to the manifestation of nonhemorrhagic-type CAA might result from mechanisms other than the direct effects of apoE on Aβ aggregation. Future studies are eagerly awaited to resolve this issue.

The pathogenesis of AD and CAA is affected by CLU [11, 36, 38]. Wilson and coworkers reported that CLU inhibits Aβ aggregation in vitro [26, 39, 42]. Consistent with our data (Fig. 5), Yerbury et al. reported that CLU significantly inhibits Aβ amyloid fibril formation at a molar ratio of CLU:Aβ =1:100 [42]. The effects of CLU on the aggregation of Aβ in vivo are controversial. While DeMattos et al. reported that CLU promotes amyloid plaque formation in vivo [5], Qi et al. reported that CLU reduces Aβ plaques as well as the severity of CAA in vivo [30]. Interestingly, DeMattos et al. reported that *apoE*^−/−^/*Clu*^−/−^ PDAPP mice had both earlier onset and marked increase of Aβ deposition, suggesting that apoE and CLU cooperatively lower the Aβ level and suppress deposition [4]. Importantly, Wojtas et al. found a marked decrease in cortical plaque deposition but an equally striking increase in CAA in the brains of APP/PS1;*Clu*^*−/−*^ mice as compared to *Clu*^+/+^ mice. They proposed that CLU facilitates Aβ clearance along the IPAD pathway by preventing binding of Aβ to cerebrovascular BMs [36]. This model is consistent with our data indicating that CLU acts as an extracellular chaperone to prevent the manifestation of CAA.

Finally, we consider how apoE and CLU inhibit amyloid formation in vitro. Based on a nucleation-dependent polymerization model [19, 23], we confirmed that apoE and CLU inhibit the early phase of Aβ aggregation (Figs. 4 and 5) as well as the seeded aggregation of Aβ amyloid fibrils (Fig. 3). Hori et al. reported that apoE interacts with Aβ protofibrils in the order of apoE2 = E3 > E4, leading to the inhibition of the conversion of Aβ protofibrils to fibrils [15]. Narayan et al. reported that CLU binds Aβ oligomers formed during the aggregation of Aβ monomers, thereby inhibiting the further growth of these oligomers into mature amyloid fibrils [26]. These reports may indicate that apoE and CLU interact with Aβ nuclei/on-pathway oligomers formed on the Matrigel-coated beads, leading to the inhibition of the successive aggregation of these species into amyloid fibrils (Figs. 4 and 5). Additionally, we previously reported that, like CLU, apoE inhibits the in vitro growth of Aβ amyloid fibrils by binding and sequestering Aβ monomers [24] (Fig. 3).

## Conclusions

We performed proteome analysis with the vessels biopsied from symptomatic, clinical CAA patients and confirmed that the expression of both apoE and CLU are significantly increased in the vessels of CAA patients as compared to non-CAA patients. Next, we used a unique in vitro model of CAA to confirm that apoE and CLU specifically inhibit the early phase of Aβ aggregation on the surface of BM-coated beads. The interaction of Aβ with vascular BMs may be a promising therapeutic target for CAA. Future studies are essential to develop the therapeutics for CAA.

## Abbreviations

AD: Alzheimer’s disease
apoE: apolipoprotein E
Aβ: amyloid-β
BM: Basement membrane
CAA: Cerebral amyloid angiopathy
CLU: Clusterin
CSF: Cerebrospinal fluid
fAβ(1–40): Aβ(1–40) amyloid fibrils
HSA: Human serum albumin
IPAD: Intramural periarterial drainage
LCM: Laser capture microdissection
LC-MS/MS: Liquid chromatography-tandem mass spectrometry
MRI: Magnetic resonance imaging
NSAF: Normalized spectral abundance factor
PBS: Phosphate buffered saline
SRPX1: Sushi repeat-containing protein 1
ThT: Thioflavin T
